# A comparative analysis of large language models versus traditional information extraction methods for real-world evidence of patient symptomatology in acute and post-acute sequelae of SARS-CoV-2

**DOI:** 10.1371/journal.pone.0323535

**Published:** 2025-05-15

**Authors:** Vedansh Thakkar, Greg M. Silverman, Abhinab Kc, Nicholas E. Ingraham, Emma K. Jones, Samantha King, Genevieve B. Melton, Rui Zhang, Christopher J. Tignanelli

**Affiliations:** 1 Department of Surgery, University of Minnesota, Minneapolis, Minnesota, United States of America; 2 Natural Language Processing/Information Extraction Program, University of Minnesota, Minneapolis, Minnesota, United States of America; 3 University of Minnesota Medical School, Minneapolis, Minnesota, United States of America; 4 Department of Pulmonary, Allergy, Critical Care, and Sleep Medicine, University of Minnesota, Minneapolis, Minnesota, United States of America; 5 Center for Learning Health Systems Sciences, University of Minnesota, Minneapolis, Minnesota, United States of America; 6 Department of Surgery, University of Washington, Seattle, Washington, United States of America; University Hospital of Padova, ITALY

## Abstract

**Background:**

Patient symptoms, crucial for disease progression and diagnosis, are often captured in unstructured clinical notes. Large language models (LLMs) offer potential advantages in extracting patient symptoms compared to traditional rule-based information extraction (IE) systems.

**Methods:**

This study compared fine-tuned LLMs (LLaMA2-13B and LLaMA3-8B) against BioMedICUS, a rule-based IE system, for extracting symptoms related to acute and post-acute sequelae of SARS-CoV-2 from clinical notes. The study utilized three corpora: UMN-COVID, UMN-PASC, and N3C-COVID. Prevalence, keyword and fairness analyses were conducted to assess symptom distribution and model equity across demographics.

**Results:**

BioMedICUS outperformed fine-tuned LLMs in most cases. On the UMN PASC dataset, BioMedICUS achieved a macro-averaged F1-score of 0.70 for positive mention detection, compared to 0.66 for LLaMA2-13B and 0.62 for LLaMA3-8B. For the N3C COVID dataset, BioMedICUS scored 0.75, while LLaMA2-13B and LLaMA3-8B scored 0.53 and 0.68, respectively for positive mention detection. However, LLMs performed better in specific instances, such as detecting positive mentions of change in sleep in the UMN PASC dataset, where LLaMA2-13B (0.79) and LLaMA3-8B (0.65) outperformed BioMedICUS (0.60). For fairness analysis, BioMedICUS generally showed stronger performance across patient demographics. Keyword analysis using ANOVA on symptom distributions across all three corpora showed that both corpus (df = 2, p < 0.001) and symptom (df = 79, p < 0.001) have a statistically significant effect on log-transformed term frequency-inverse document frequency (TF-IDF) values such that corpus accounts for 52% of the variance in log_tfidf values and symptom accounts for 35%.

**Conclusion:**

While BioMedICUS generally outperformed the LLMs, the latter showed promising results in specific areas, particularly LLaMA3-8B, in identifying negative symptom mentions. However, both LLaMA models faced challenges in demographic fairness and generalizability. These findings underscore the need for diverse, high-quality training datasets and robust annotation processes to enhance LLMs’ performance and reliability in clinical applications.

## Introduction

Patient symptoms are critical to understanding disease progression and diagnosis [1,2]. They offer both diagnostic and prognostic capacity [2]. Structured electronic health record (EHR) data rarely capture patient symptoms, leaving them embedded within unstructured sections of clinical notes such as the history of present illness (HPI) [2]. Thus, natural language processing (NLP) solutions that can reliably extract this information from clinical notes are needed to facilitate their integration into downstream applications. This study provides a comprehensive exploration of using large language models (LLMs) for classification tasks in NLP for symptom extraction from unstructured clinical notes. It evaluates their performance against rule-based information extraction (IE) methods, particularly those reliant on the unified medical language system (UMLS). By demonstrating the comparable effectiveness of LLMs to rule-based IE methods and emphasizing their inherent advantages, such as handling concept synonymy without extensive manual intervention, this study envisions a future where labor-intensive lexica creation by subject matter experts (SMEs) becomes obsolete.

The UMLS metathesaurus integrates biomedical concepts from diverse vocabularies into concept unique identifiers (CUIs) [3]. With over 4 million concepts grouped into 15 semantic groups, 133 semantic types, and 54 semantic relationships, the UMLS facilitates various applications, including IE [3–5]. PASCLex is a comprehensive lexicon of UMLS concepts and their synonyms related to Post-Acute Sequelae of SARS-CoV-2 (PASC, also known as “Long COVID”) symptoms that was created by analyzing a large corpus of clinical notes [6]. PASCLex aims to improve the identification and analysis of symptoms beyond those specific to PASC. Clinical NLP systems like The BioMedical Information Collection and Understanding System (BioMedICUS), developed by the University of Minnesota’s Natural Language Processing/Information Extraction Program, leverages the UMLS for large-scale extensive text analysis, enabling annotation of concepts and their negations, sentence boundaries, and note section headers [7–9].

LLMs have the potential to autonomously glean relevant features from data, bypassing the laborious and expertise-dependent manual feature engineering process [10]. Moreover, they adeptly navigate intricate linguistic structures like long-distance dependencies, anaphora, and ambiguity, thereby augmenting accuracy in IE tasks [11]. By fine-tuning, LLMs swiftly acclimate to diverse domains and IE tasks, requiring minimal additional training data, thus rendering them versatile for myriad applications [12]. Given that LLMs continue to improve and become more accessible, they are poised to play a significant role in the future of IE.

### Background on symptomatology and objective

Diagnostic challenges occur daily in clinical practice, particularly in primary care, where, for example, fatigue is a common symptom among many diseases and in isolation may not provide significant predictive power. Predictive models that use a patient’s presenting symptoms to support clinical decisions can help reduce diagnostic errors. However, for these systems to be accurate, the information related to patient symptomatology, which most often exists in unstructured data, must be reliable. Studies have shown that NLP methods using patient symptoms mapped to UMLS concepts can increase efficacy for predictive modeling in various contexts, including better colorectal cancer prediction [13] enhanced influenza prediction [14], and detection of surgical complications [15].

Recognizing the worldwide burden of PASC and the need for rapid diagnosis, in 2020, our team developed an NLP system as part of an artificial intelligence (AI) automation pipeline that could autonomously extract acute COVID-19 and PASC symptoms from longitudinal clinical notes at scale across a network of 12 Midwest U.S. hospitals and 60 primary care clinics [2]. Both structured clinical data and NLP-based features engineered from patient symptoms extracted at scale from clinical notes were used to populate a registry of COVID-19 patients [2,16]. We utilized our AI automation pipeline for monitoring PASC at the patient level for over 83,850 patients diagnosed with acute COVID-19, showing that these data could effectively surveil post-COVID patients to accurately identify patients suspected of having PASC [17]. This previous methodology utilized both rule-based and UMLS-based approaches for autonomous symptom extraction within our AI pipeline [2,16]. Over the past 4 years, LLMs have been developed and are rapidly becoming the status quo in general purpose NLP. However, their role is less certain within biomedical applications, and previous work has shown that bidirectional encoder representations from transformers (BERT) [18] based approach may outperform LLMs for certain specific biomedical tasks [19].

The objective of this study was to fine-tune LLMs (LLaMA2-13B and LlaMA3-18B) [20]for extraction of patient symptoms from unstructured clinical notes and demonstrate the comparable effectiveness of the fine-tuned LLMs to rule-based IE methods, such as BioMedICUS. We also aimed to explore the possibility of integrating the fine-tuned LLMs into the BioMedICUS pipeline. Notably, BioMedICUS stands out for its capability to handle notes in rich text format (RTF), a feature not commonly found in other NLP systems, which made it an appealing choice for our purposes [8].

## Materials and methods

This study utilized three different corpora of ground truth labeled clinical notes for fine-tuning and testing of the LLMs and for comparison with BioMedICUS. University of Minnesota (UMN) COVID, accessed in October 2020, a corpus of emergency department (ED) visit notes for patients diagnosed with acute COVID-19 as described in [2] and UMN PASC, accessed in September 2021, a corpus of ED and outpatient (OP) visits for patients diagnosed with acute COVID-19 followed by lingering symptoms were provided by UMN/MHealth Fairview and were manually annotated by three subject matter experts with an inter rater reliability of 0.68 computed using Fleiss’ kappa [21]. The National COVID Cohort Collaborative (N3C) COVID, a fully de-identified corpus of notes for patients diagnosed with acute COVID-19 was provided by the Mayo Clinic in June 2021. These three corpora were used because they were available with ground truth annotations for symptom identification and offered an opportunity for both internal and external validation of the fine-tuned LLM. All the authors had access to information that could identify individual participants during and after the data collection. A summary of each corpus is presented in Table 1.

**Table 1:** Summary of corpora with note counts utilized for LLM development.

Corpus	Source	Note count
UMN PASC	Outpatient (OP)	387
	Emergency Department (ED)	84
UMN COVID	Emergency Department (ED)	46
N3C COVID	Not Available (NA)	148

For UMN COVID, criteria for inclusion and manual curation methods of this corpus are outlined in (2). For UMN PASC, the inclusion criteria for the UMN PASC corpus mirror those of the UMN COVID corpus, with the following added criteria: patients must have (1) no baseline symptoms, (2) presented within a timeframe exceeding 30 days from their initial COVID-19 infection, and (3) at least one new or residual symptom [17]. Annotation guidelines instructed annotators to identify symptoms and their negations within the HPI section and other relevant sections. Clinical notes were randomly sampled from encounters at least 60 days after diagnosis with COVID-19. N3C COVID corpus is a fully de-identified, manually annotated set of 148 unstructured notes across a selection of 5 inpatients and 5 outpatients with research authorizations as described by [16]. This corpus’s inclusion criteria were: (1) notes documented within two weeks before and four weeks after the lab order date of the first positive COVID-19 result, and (2) notes that contained more than 1000 characters. Up to 20 notes from each patient were included in this corpus [22,23]. Demographics of the three corpora are presented in Table 2.

**Table 2:** Demographics of various corpora.

Corpus	Number of Patients	Median age in years, IQR (Q1, Q3)	Male %	Racial Distribution and Ethnicity %
UMN PASC	476	53.10, (38.00, 67.90)	58.00%	5.00% Asian
				11.90% Black
				3.36% Hispanic
				15.74% Others*
				64.00% White
UMN COVID	46	54.68, (39.00, 66.64)	54.00%	9.09% Asian
				27.27% Black
				4.54% Hispanic
				7.10% Others*
				52.00% White
N3C COVID**	148	Not Available (NA)	NA	NA

*Others include Declined races. **N3C COVID dataset is de-identified

For prevalence and keyword analyses, we examined how document format (PASC) might influence model training by analyzing symptoms as keywords [24]. BioMedICUS’ annotations were converted to a normalized format using the PASCLex dictionary to allow for keyword comparisons between corpora used in this study. Unlike the previous study of Wang, et. al. [6] that simply ranked terms by how often symptoms appeared in their corpus, this study used the Term Frequency-Inverse Document Frequency (TF-IDF) statistic to analyze how symptoms (as keywords) were distributed across documents within each corpus. This approach provides a more nuanced picture than just counting total occurrences of a word in a corpus: A keyword’s frequency (TF) measures how often that term appears in a document, while a term’s inverse document frequency (IDF) measures how important that term as a keyword is across all documents in the corpus. The product of TF and IDF thus helps identify keywords that are more meaningful or relevant to specific documents across the corpus (24). To compare keyword usage between corpora we performed an analysis of variance (ANOVA) test followed by *ad hoc* tests to determine which factors in our model were significantly different [24–26]. Finally, we calculated effect size (η²) to assess the magnitude of these differences [24]. S1 Table shows the top 10 extracted keywords ranked by TF-IDF and prevalence for normalized symptoms mapped to the PASCLex dictionary. S2 Table represents an example input data for ANOVA. S3 Table shows an example data frame used for the prevalence and keyword analysis. Actual data wasn’t shown due to HIPAA compliance. The TF-IDF code and the ANOVA/effect size sample code is also provided in the supporting information.

We fine-tune LLaMA2-13B and LLaMA3-8B models using clinical notes from 522 patients: 46 with acute COVID-19 and 476 with PASC. The median age of the patients was 53.89 years, with 55% male. We divided the PASC corpus into three sets: 65% for training, 4% for validation, and 31% for hold-out testing. All COVID-19 patient notes were used in the training set because they were annotated by a single SME without a consensus process.. To test the model’s generalizability, we included all patients from the N3C COVID corpus in our hold-out test set. This resulted in 351 patients in the training set, 20 in the validation set, and 299 in the hold-out test set. The demographic characteristics of each of these distinct corpora are delineated in Table 3.

**Table 3:** Breakdown of distinct corpora for LLM fine-tuning, validation, and hold-out testing.

Corpora	Number of Patients	Median age in years, IQR (Q1, Q3)	Male %	Racial Distribution and Ethnicity %
Training	351	53.89, (38.00, 67.00)	55.00%	7.00% Asian
				19.50% Black
				3.90% Hispanic
				11.60% Others*
				58.00% White
Validation	20	53.50, (38.00, 67.00)	56.00%	6.00% Asian
				18.00% Black
				4.00% Hispanic
				12.00% Others*
				60.00% White
Hold-out Test**	299	53.00, (38.00, 67.00)	56.00%	6.5% Asian
				20% Black
				4.00% Hispanic
				11.5% Others*
				58% White

*Others include Declined races.

**The demographics of the test set include information about UMN PASC patients only, since the N3C COVID data are de-identified.

For model validation, metrics including macro-averaged recall, precision, and F1-score were calculated to determine the models’s performance in applying extracted symptoms for labeling of clinical notes. To assess the LLMs’ and BioMedICUS’ fairness across race and gender, their performance was evaluated on the UMN PASC hold-out test set for which data on race or ethnicity and gender were available. We performed an error analysis to determine sources of false negatives (FN) for BioMedICUS and the LLMs used in this study, we examined a few symptoms in which the false negative rate (FNR) was: (1) high for the LLMs, but not BioMedICUS (**fever** for N3C corpus); (2) high for BioMedICUS, but not for the LLMs (**diarrhea** for UMN PASC corpus); and (3) both high for BioMedICUS and LLMs (**chest pain** for N3C corpus).

### Fine-tuned versus un-fine-tuned models for symptom classification

To assess the impact of fine-tuning on structured symptom extraction, we evaluated both the un-fine-tuned LLaMA2-13B model and our fine-tuned variant. The primary goal was to determine whether fine-tuning improves symptom classification performance, output consistency, reduces post-processing effort, and enhances integration into clinical workflows.

#### Evaluation of un-fine-tuned model.

We experimented with multiple prompting strategies, including zero-shot and few-shot prompting, to instruct the un-fine-tuned LLaMA2-13B model to classify 14 symptoms of interest as present (1), absent (0), or negated (-1). A representative prompt used in our evaluation was:

*System prompt: “You are a medical language model trained to accurately analyze and extract information from clinical notes. Ensure you correctly identify and differentiate between reported symptoms and negated symptoms. Pay attention to medical terminology and context to provide precise and reliable information.”*

*User prompt: “Classify the symptoms given in the provided clinical note as present, absent, or negated. Positive symptoms are the symptoms that the patient has or recently had. Negative symptoms are the symptoms that are explicitly denied or negated (e.g., denies fever). List the positive and negative symptoms in a JSON format. Here is the clinical note: {clinical_note}. Please classify only the symptoms given in the following list: {symptom_list}.”*

The model’s output was analyzed for adherence to structured formatting, consistency in terminology, and alignment with predefined symptom categories.

#### Fine-tuning procedure.

To extract symptoms from clinical notes, we fine-tuned the LLaMA2-13B and LLaMA3-8B models for a classification task. We selected LLaMA models due to their open-source availability, strong performance on medical text processing tasks, and ability to be fine-tuned for specialized applications without licensing constraints. Proprietary models such as Azure HIPAA-Compliant GPT-4 may offer superior performance in some contexts; however, their lack of transparency, higher deployment costs, and restricted adaptability for research purposes made them less suitable for our study. Furthermore, institutional policies at the University of Minnesota prohibit the use of cloud-based LLMs, reinforcing our decision to use locally hosted models. We fine-tuned LLaMA2-13B and LLaMA3-8B models, as they provided a balance between computational feasibility and performance. Larger models such as LLaMA 70B or 405B were not used due to resource constraints.

Our fine-tuned models classify symptoms into three categories: “present,” “negated,” and “absent.” The classification task was framed as a multi-label learning problem, where each symptom in a clinical note was assigned one of the three categories based on its context. The training dataset consisted of annotated clinical notes, with symptom labels assigned by domain experts. Our fine-tuning approach employed a parameter efficient fine-tuning technique. The LLaMA models consist of multiple transformer layers, with the final layer being crucial for task-specific adaptation. In our implementation, we primarily focused on modifying the final layer while keeping earlier layers frozen to retain general language understanding while adapting to the clinical classification task. The earlier layers of the base LLaMA models were frozen to preserve general language representations while allowing the output layer to be fine-tuned for classification. The output classification layer consists of a linear transformation with 42 output neurons, corresponding to 14 symptoms classified across three labels. Each symptom is assigned a classification label: “-1” for negation (e.g., “denied headache”), “0” for absence, and “1” for presence.

Our models were fine-tuned for approximately 50 epochs, using a batch size of 4 and a gradient clipping value of 0.3 to stabilize updates. We applied a learning rate of 2e-4 with a polynomial decay schedule and warm-up to enhance convergence. Additionally, a weight decay of 1e-3 was used to prevent overfitting. The AdamW optimizer was utilized with default settings, as it demonstrated optimal performance during validation.

## Results

### Prevalence and keyword analysis

For prevalence and keyword analysis, each cohort in the corpora used in this study had its top 10 extracted keywords ranked by TF-IDF for normalized symptoms mapped to PASCLex, as summarized in S1 Table. Keywords ranked by prevalence are also summarized in S1 Table. As presented in S4 Table, hypothesis testing using ANOVA on both corpus (df = 2, p < 0.001) and symptom (df = 79, p < 0.001) have statistically significant effects on log-transformed TF-IDF values suggesting that TF-IDF values are strongly influenced by the source of the text (corpus) and the type of symptom being described. Results of post hoc testing presented in S5 Table revealed significant differences in TF-IDF values between UMN COVID and UMN PASC (mean difference = 2.57, p < 0.001), UMN COVID and N3C COVID (mean difference = 1.06 p = 0.0001), and UMN PASC and N3C COVID (mean difference = –1.89, p < 0.001). These results indicate that the textual representation of symptoms as keywords differs notably between corpora. Furthermore, S6 Table showed there were large effects for both corpus (η² = 0.52) and symptoms (η² = 0.35).

### Fine-tuned LLMs versus BioMedICUS

To validate the fine-tuned LLMs’ performance and compare the results with BioMedICUS, we used the hold-out test set to evaluate extraction performance. Table 4 displays symptom-wise macro-average scores of performance metrics calculated over UMN PASC symptoms.

**Table 4:** Macro-averaged metrics with 95% confidence intervals for evaluation of BioMedICUS’, LLaMA2-13B, and LLaMA3-8B extraction performance in positive and negative symptom mentions for UMN PASC.

	Positive Symptom Mentions			Negative Symptom Mentions		
Symptoms and Models	Precision	Recall	F1-score	Precision	Recall	F1-score
bone or joint pain						
BioMedICUS	0.56(0.53-0.58)	0.61(0.56-0.65)	0.37(0.33-0.41)	0.59(0.57-0.62)	0.65(0.61-0.69)	0.45(0.40-0.49)
LLaMA2	0.56(0.50-0.63)	0.66(0.50-0.83)	0.57(0.47-0.66)	0.54(0.45-0.63)	0.54(0.46-0.62)	0.54(0.45-0.62)
LLaMA3	0.58(0.52-0.65)	0.74(0.58-0.89)	0.57(0.48-0.66)	0.70(0.57-0.83)	0.63(0.54-0.73)	0.65(0.55-0.76)
mental status changes						
BioMedICUS	0.61(0.56-0.66)	0.77(0.67-0.87)	0.64(0.57-0.70)	0.57(0.53-0.62)	0.80(0.66-0.91)	0.59(0.53-0.65)
LLaMA2	0.58(0.46-0.82)	0.55(0.48-0.69)	0.56(0.47-0.71)	0.48(0.46-0.49)	0.49(0.47-0.50)	0.48(0.47-0.49)
LLaMA3	0.47(0.46-0.49)	0.49(0.47-0.50)	0.48(0.47-0.49)	0.48(0.46-0.49)	0.48(0.47-0.50)	0.48(0.47-0.49)
chest pain/palpitations						
BioMedICUS	0.70(0.63-0.77)	0.69(0.62-0.76)	0.69(0.63-0.76)	0.85(0.81-0.89)	0.73(0.69-0.77)	0.76(0.71-0.80)
LLaMA2	0.74(0.60-0.89)	0.73(0.59-0.86)	0.74(0.61-0.85)	0.78(0.68-0.88)	0.67(0.59-0.75)	0.70(0.60-0.78)
LLaMA3	0.57(0.53-0.61)	0.71(0.61-0.78)	0.47(0.38-0.54)	0.84(0.76-0.90)	0.87(0.81-0.92)	0.85(0.78-0.91)
cough						
BioMedICUS	0.83(0.78-0.87)	0.90(0.86-0.93)	0.85(0.81-0.89)	0.78(0.74-0.81)	0.83(0.80-0.87)	0.78(0.74-0.82)
LLaMA2	0.68(0.57-0.78)	0.64(0.55-0.74)	0.65(0.55-0.74)	0.70(0.60-0.80)	0.66(0.58-0.75)	0.68(0.58-0.76)
LLaMA3	0.74(0.66-0.82)	0.83(0.75-0.90)	0.76(0.68-0.84)	0.79(0.70-0.86)	0.78(0.69-0.86)	0.78(0.70-0.85)
diarrhea						
BioMedICUS	0.72(0.65-0.79)	0.81(0.73-0.89)	0.75(0.68-0.82)	0.91(0.87-0.95)	0.94(0.91-0.97)	0.92(0.89-0.95)
LLaMA2	0.69(0.58-0.81)	0.85(0.70-0.97)	0.74(0.60-0.85)	0.65(0.54-0.75)	0.64(0.54-0.73)	0.64(0.54-0.73)
LLaMA3	0.70(0.57-0.82)	0.85(0.68-0.97)	0.75(0.59-0.86)	0.84(0.73-0.94)	0.75(0.65-0.86)	0.79(0.68-0.88)
shortness of breath						
BioMedICUS	0.73(0.69-0.76)	0.84(0.81-0.88)	0.75(0.70-0.79)	0.78(0.74-0.81)	0.81(0.77-0.84)	0.77(0.73-0.81)
LLaMA2	0.70(0.61-0.79)	0.76(0.65-0.86)	0.72(0.62-0.80)	0.66(0.56-0.75)	0.62(0.55-0.69)	0.63(0.54-0.70)
LLaMA3	0.62(0.56-0.69)	0.73(0.63-0.82)	0.61(0.52-0.70)	0.59(0.49-0.68)	0.57(0.49-0.64)	0.57(0.48-0.65)
fatigue BioMedICUS	0.78(0.74-0.82)	0.87(0.83-0.91)	0.81(0.77-0.85)	0.61(0.56-0.65)	0.80(0.69-0.89)	0.64(0.57-0.70)
LLaMA2	0.70(0.61-0.79)	0.76(0.65-0.86)	0.72(0.62-0.80)	0.59(0.49-0.71)	0.68(0.46-0.87)	0.62(0.48-0.75)
LLaMA3	0.70(0.63-0.77)	0.86(0.80-0.91)	0.71(0.63-0.79)	0.54(0.48-0.61)	0.63(0.42-0.83)	0.54(0.45-0.64)
fever						
BioMedICUS	0.64(0.60-0.68)	0.82(0.77-0.87)	0.66(0.61-0.71)	0.89(0.86-0.92)	0.89(0.86-0.92)	0.89(0.86-0.92)
LLaMA2	0.57(0.52-0.65)	0.69(0.54-0.82)	0.57(0.48-0.67)	0.62(0.54-0.71)	0.61(0.53-0.69)	0.61(0.52-0.69)
LLaMA3	0.59(0.54-0.64)	0.78(0.69-0.85)	0.54(0.46-0.63)	0.76(0.69-0.82)	0.76(0.69-0.82)	0.76(0.69-0.82)
headaches BioMedICUS	0.72(0.67-0.78)	0.87(0.82-0.92)	0.77(0.71-0.82)	0.82(0.77-0.88)	0.92(0.88-0.96)	0.86(0.81-0.91)
LLaMA2	0.72(0.59-0.87)	0.72(0.59-0.86)	0.72(0.59-0.84)	0.67(0.55-0.80)	0.67(0.55-0.80)	0.67(0.55-0.78)
LLaMA3	0.64(0.57-0.71)	0.85(0.76-0.90)	0.65(0.55-0.74)	0.66(0.56-0.75)	0.71(0.59-0.83)	0.68(0.57-0.77)
changes in sleep BioMedICUS	0.58(0.53-0.62)	0.79(0.66-0.89)	0.60(0.53-0.66)	0.60(0.51-0.71)	0.61(0.51-0.74)	0.60(0.51-0.71)
LLaMA2	0.86(0.49-1.00)	0.75(0.50-1.00)	0.79(0.49-0.96)	0.50(0.49-1.00)	0.50(0.50-1.00)	0.50(0.49-1.00)
LLaMA3	0.61(0.52-0.72)	0.79(0.57-0.97)	0.65(0.52-0.77)	0.50(0.49-1.00)	0.50(0.49-1.00)	0.50(0.49-1.00)
nausea/vomiting						
BioMedICUS	0.68(0.64-0.72)	0.87(0.83-0.91)	0.71(0.66-0.76)	0.87(0.84-0.91)	0.91(0.88-0.93)	0.89(0.85-0.92)
LLaMA2	0.63(0.54-0.73)	0.72(0.58-0.86)	0.65(0.54-0.76)	0.69(0.59-0.78)	0.68(0.59-0.77)	0.68(0.59-0.77)
LLaMA3	0.62(0.56-0.68)	0.80(0.69-0.89)	0.62(0.53-0.71)	0.81(0.72-0.89)	0.81(0.72-0.89)	0.81(0.72-0.88)
nasal congestion						
BioMedICUS	0.68(0.62-0.74)	0.86(0.78-0.93)	0.73(0.65-0.79)	0.84(0.77-0.90)	0.86(0.79-0.92)	0.85(0.79-0.90)
LLaMA2	0.63(0.48-0.80)	0.63(0.48-0.77)	0.63(0.48-0.75)	0.69(0.54-0.86)	0.63(0.53-0.76)	0.65(0.52-0.77)
LLaMA3	0.58(0.52-0.66)	0.71(0.54-0.87)	0.59(0.50-0.69)	0.64(0.50-0.79)	0.60(0.50-0.72)	0.61(0.50-0.72)
sore throat						
BioMedICUS	0.67(0.61-0.73)	0.93(0.88-0.97)	0.73(0.66-0.80)	0.90(0.84-0.94)	0.86(0.80-0.92)	0.88(0.82-0.92)
LLaMA2	0.56(0.48-0.67)	0.60(0.45-0.80)	0.57(0.47-0.69)	0.63(0.47-0.80)	0.60(0.48-0.74)	0.62(0.47-0.74)
LLaMA3	0.56(0.51-0.61)	0.75(0.56-0.91)	0.55(0.46-0.63)	0.71(0.59-0.85)	0.76(0.62-0.91)	0.74(0.60-0.85)
loss of smell and taste						
BioMedICUS	0.77(0.66-0.90)	0.70(0.61-0.81)	0.73(0.63-0.82)	0.74(0.57-0.90)	0.67(0.55-0.80)	0.70(0.56-0.82)
LLaMA2	0.98(0.46-0.99)	0.56(0.50-0.71)	0.60(0.48-0.79)	0.74(0.48-1.00)	0.66(0.49-1.00)	0.69(0.49-1.00)
LLaMA3	0.65(0.53-0.76)	0.77(0.57-0.96)	0.68(0.54-0.80)	0.67(0.50-0.85)	0.98(0.48-0.99)	0.74(0.49-0.91)

As presented in Table 4, for UMN PASC positive symptoms, BioMedICUS consistently outperformed LLaMA2-13B and LLaMA3-8B in most cases, particularly **mental status changes, cough, shortness of breath, fatigue, fever, headaches, nausea/vomiting, nasal congestion, sore throat,** and **loss of smell and taste**, where it achieved the highest F1-scores. LLaMA2-13B excelled in **chest pain/palpitations** and **changes in sleep**, surpassing both BioMedICUS and LLaMA3-8B. For UMN PASC negative symptoms, BioMedICUS demonstrated superior performance in most cases, leading in **mental status changes, cough, diarrhea, shortness of breath, fever, headaches, nausea/vomiting, nasal congestion, sore throat,** and **loss of smell and taste**. LLaMA3-8B outperformed both models for **bone or joint pain**, and it also showed strong results for **chest pain/palpitations**, achieving the highest recall and F1-score.

Tables 5 displays symptom-wise macro-average scores of performance metrics calculated over N3C COVID symptoms.

**Table 5:** Macro-averaged metrics with 95% confidence intervals for evaluation of BioMedICUS’, LLaMA2-13B, and LLaMA3-8B extraction performance in positive and negative symptom mentions for N3C COVID.

	Positive Symptom Mentions			Negative Symptom Mentions		
Symptoms and Models	Precision	Recall	F1-score	Precision	Recall	F1-score
bone or joint pain						
BioMedICUS	0.54(0.51-0.59)	0.75(0.70-0.80)	0.41(0.34-0.50)	0.50(0.47-0.54)	0.53(0.33-0.87)	0.45(0.39-0.52)
LLaMA2	0.50(0.47-0.54)	0.48(0.35-0.73)	0.45(0.41-0.52)	0.49(0.47-0.50)	0.46(0.43-0.48)	0.47(0.46-0.49)
LLaMA3	0.58(0.49-0.71)	0.67(0.46-0.97)	0.60(0.47-0.75)	0.64(0.49-0.83)	0.82(0.48-0.99)	0.69(0.49-0.87)
mental status changes						
BioMedICUS	0.76(0.53-0.97)	0.68(0.52-0.86)	0.71(0.52-0.86)	0.66(0.48-1.00)	0.66(0.48-1.00)	0.66(0.48-0.90)
LLaMA2	0.61(0.49-0.74)	0.62(0.49-0.75)	0.61(0.49-0.73)	0.49(0.48-0.50)	0.49(0.47-0.50)	0.49(0.48-0.50)
LLaMA3	0.98(0.96-0.99)	0.77(0.62-0.91)	0.84(0.67-0.95)	0.49(0.48-0.50)	0.49(0.48-0.50)	0.49(0.48-0.50)
chest pain/palpitations						
BioMedICUS	0.56(0.44-0.79)	0.54(0.47-0.67)	0.54(0.46-0.69)	0.89(0.65-1.00)	0.78(0.57-1.00)	0.82(0.59-0.96)
LLaMA2	0.51(0.45-0.58)	0.51(0.44-0.63)	0.51(0.45-0.59)	0.47(0.45-0.49)	0.47(0.45-0.49)	0.47(0.46-0.48)
LLaMA3	0.58(0.52-0.66)	0.74(0.57-0.91)	0.60(0.49-0.70)	0.58(0.52-0.67)	0.78(0.58-0.94)	0.60(0.49-0.71)
cough						
BioMedICUS	0.90(0.82-0.96)	0.92(0.84-0.97)	0.91(0.83-0.96)	0.68(0.47-0.98)	0.65(0.48-0.88)	0.66(0.48-0.83)
LLaMA2	0.55(0.47-0.63)	0.56(0.46-0.66)	0.55(0.46-0.64)	0.51(0.47-0.58)	0.53(0.42-0.70)	0.51(0.45-0.60)
LLaMA3	0.83(0.74-0.91)	0.84(0.76-0.92)	0.84(0.75-0.91)	0.62(0.53-0.71)	0.86(0.67-0.96)	0.65(0.52-0.76)
diarrhea						
BioMedICUS	0.91(0.83-0.97)	0.89(0.80-0.97)	0.90(0.82-0.96)	0.83(0.70-0.93)	0.87(0.74-0.97)	0.85(0.73-0.93)
LLaMA2	0.59(0.52-0.67)	0.64(0.53-0.75)	0.60(0.51-0.69)	0.51(0.45-0.58)	0.52(0.41-0.64)	0.50(0.43-0.59)
LLaMA3	0.88(0.80-0.96)	0.91(0.83-0.98)	0.90(0.81-0.96)	0.74(0.61-0.87)	0.76(0.61-0.89)	0.75(0.61-0.86)
shortness of breath						
BioMedICUS	0.77(0.70-0.84)	0.80(0.72-0.87)	0.76(0.68-0.83)	0.68(0.57-0.81)	0.71(0.59-0.84)	0.70(0.58-0.80)
LLaMA2	0.61(0.52-0.71)	0.59(0.51-0.68)	0.60(0.51-0.68)	0.53(0.45-0.62)	0.54(0.44-0.65)	0.53(0.45-0.63)
LLaMA3	0.71(0.65-0.78)	0.78(0.70-0.84)	0.72(0.63-0.79)	0.56(0.49-0.65)	0.60(0.48-0.73)	0.57(0.48-0.67)
fatigue						
BioMedICUS	0.80(0.68-0.91)	0.83(0.71-0.94)	0.82(0.70-0.91)	0.62(0.50-1.00)	0.99(0.47-1.00)	0.69(0.49-1.00)
LLaMA	0.54(0.48-0.62)	0.57(0.45-0.71)	0.54(0.46-0.64)	0.50(0.49-0.50)	0.48(0.46-0.50)	0.49(0.48-0.50)
LLaMA3	0.68(0.58-0.78)	0.78(0.64-0.90)	0.71(0.60-0.81)	0.52(0.50-0.57)	0.93(0.41-0.95)	0.51(0.45-0.60)
fever						
BioMedICUS	0.85(0.75-0.94)	0.83(0.73-0.93)	0.84(0.74-0.92)	0.90(0.81-0.97)	0.88(0.79-0.96)	0.89(0.80-0.96)
LLaMA2	0.59(0.51-0.67)	0.62(0.52-0.74)	0.59(0.50-0.68)	0.59(0.51-0.68)	0.63(0.52-0.75)	0.60(0.51-0.69)
LLaMA3	0.59(0.53-0.64)	0.67(0.57-0.76)	0.53(0.45-0.62)	0.65(0.57-0.72)	0.71(0.61-0.81)	0.66(0.57-0.74)
headaches						
BioMedICUS	0.82(0.58-0.99)	0.78(0.56-0.99)	0.80(0.57-0.94)	0.80(0.62-0.99)	0.84(0.65-1.00)	0.82(0.63-0.95)
LLaMA2	0.53(0.47-0.61)	0.58(0.42-0.79)	0.53(0.45-0.64)	0.51(0.46-0.60)	0.53(0.43-0.70)	0.51(0.45-0.62)
LLaMA3	0.57(0.50-0.66)	0.72(0.51-0.93)	0.59(0.48-0.71)	0.60(0.52-0.69)	0.79(0.59-0.94)	0.62(0.51-0.74)
changes in sleep						
BioMedICUS	0.67(0.50-1.00)	0.99(0.48-1.00)	0.75(0.49-1.00)	1.00(0.49-1.00)	0.75(0.50-1.00)	0.83(0.49-1.00)
LLaMA2	0.50(0.49-1.00)	0.50(0.50-1.00)	0.50(0.49-1.00)	0.49(0.48-1.00)	0.50(0.50-1.00)	0.50(0.49-1.00)
LLaMA3	0.50(0.49-1.00)	0.50(0.50-1.00)	0.50(0.49-1.00)	0.49(0.48-1.00)	0.50(0.50-1.00)	0.50(0.49-1.00)
nausea/vomiting						
BioMedICUS	0.83(0.74-0.93)	0.85(0.76-0.94)	0.84(0.75-0.92)	0.76(0.64-0.88)	0.77(0.66-0.89)	0.77(0.65-0.86)
LLaMA2	0.48(0.42-0.55)	0.47(0.39-0.56)	0.47(0.40-0.55)	0.47(0.41-0.56)	0.48(0.43-0.54)	0.47(0.42-0.55)
LLaMA3	0.68(0.60-0.75)	0.76(0.66-0.85)	0.69(0.60-0.78)	0.69(0.60-0.77)	0.79(0.68-0.89)	0.72(0.61-0.80)
nasal congestion						
BioMedICUS	0.49(0.48-0.50)	0.49(0.48-0.50)	0.49(0.48-0.50)	0.75(0.50-1.00)	1.00(0.49-1.00)	0.83(0.49-1.00)
LLaMA2	0.49(0.48-0.50)	0.43(0.40-0.46)	0.46(0.44-0.47)	0.50(0.49-0.50)	0.48(0.46-0.49)	0.49(0.48-0.50)
LLaMA3	0.49(0.48-1.00)	0.50(0.50-1.00)	0.50(0.49-1.00)	0.50(0.49-1.00)	0.50(0.50-1.00)	0.50(0.49-1.00)
sore throat						
BioMedICUS	0.86(0.67-1.00)	0.99(0.97-1.00)	0.91(0.74-1.00)	0.99(0.48-1.00)	0.75(0.50-1.00)	0.83(0.49-1.00)
LLaMA2	0.51(0.47-0.57)	0.54(0.42-0.75)	0.50(0.45-0.59)	0.49(0.47-0.50)	0.47(0.45-0.49)	0.48(0.47-0.49)
LLaMA3	0.78(0.60-0.94)	0.99(0.97-1.00)	0.85(0.65-0.96)	0.75(0.57-0.93)	0.99(0.97-1.00)	0.83(0.61-0.96)
loss of smell and taste						
BioMedICUS	0.81(0.59-0.99)	0.74(0.55-0.98)	0.77(0.56-0.91)	0.66(0.48-1.00)	0.66(0.48-1.00)	0.66(0.48-0.90)
LLaMA2	0.47(0.45-0.49)	0.49(0.47-0.50)	0.48(0.47-0.49)	0.49(0.48-0.50)	0.49(0.48-0.50)	0.49(0.48-0.50)
LLaMA3	0.81(0.47-0.99)	0.62(0.49-0.81)	0.67(0.48-0.85)	0.64(0.49-0.83)	0.82(0.48-0.99)	0.69(0.49-0.87)

As presented in Table 5, for N3C COVID positive symptoms, BioMedICUS was the top-performing model for **cough, diarrhea, shortness of breath, fatigue, fever, headaches, nausea/vomiting, sore throat,** and **loss of smell and taste**, consistently achieving the highest F1-scores. LLaMA3-8B excelled in **mental status changes,** Bone or joint pain, and **chest pain/palpitations**. All three models performed similarly for **changes in sleep** and **nasal congestion**, with relatively lower scores. For N3C COVID negative symptoms, LLaMA3-8B demonstrated the highest performance for **bone or joint pain**, outperforming BioMedICUS and LLaMA2-13B in all metrics. BioMedICUS was the best for **mental status changes** and **chest pain/palpitations**, achieving the highest precision and recall. LLaMA3-8B also excelled in **cough classification**, while BioMedICUS led in **diarrhea** detection. Notably, LLaMA2-13B performed best in **fatigue, fever, headaches,** and **sore throat**, achieving the highest F1-scores in these cases.

For fairness analysis, as seen in Table 6, BioMedICUS consistently outperformed the other models across patient demographics, including gender (male, female) and racial groups (Asian, Black, White, and Other). Fine-tuned LLaMA2-13B showed the lowest recall, precision, and F1-scores across both positive and negative symptom mentions, indicating a limited ability to handle nuanced patient demographic data. In contrast, Fine-tuned LLaMA3-8B demonstrated improved performance, especially for the White racial group, but still lagged behind BioMedICUS. BioMedICUS exhibited the highest F1-scores in nearly all categories, particularly excelling in negative symptom mentions, suggesting that domain-specific models hold a significant advantage in clinical contexts.

**Table 6:** Macro-averaged metrics for evaluation of BioMedICUS’, LLaMA2-13B, and LLaMA3-8B equity for race and gender in positive (+) and negative (-) symptom mentions for UMN PASC.

Model	Category	r*, p*, f1* (+)	r, p, f1 (-)
BioMedICUS	Male Female	0.82, 0.71, 0.76 0.80, 0.67, 0.73	**0.80, 0.76, 0.77** **0.78, 0.74, 0.76**
	Asian Black Other** White	0.75, 0.65, 0.69 0.80, 0.68, 0.73 0.84, 0.70, 0.76 0.85, 0.73, 0.78	**0.75, 0.70, 0.72** **0.79, 0.74, 0.76** 0.81, 0.76, 0.78 0.83, 0.80, 0.81
LLaMA2	Male Female	0.69, 0.71, 0.70 0.67, 0.66, 0.66	0.62, 0.65, 0.63 0.61, 0.62, 0.63
	Asian Black Other** White	0.64, 0.65, 0.64 0.67, 0.66, 0.66 0.70, 0.70, 0.70 0.70, 0.72, 0.71	0.58, 0.62, 0.60 0.60, 0.64, 0.61 0.62, 0.65, 0.63 0.63, 0.66, 0.64
LLaMA3	Male Female	**0.85, 0.73, 0.79** **0.82, 0.69, 0.75**	0.79, 0.75, 0.74 0.77, 0.73, 0.75
	Asian Black Other** White	0.74, 0.63, 0.67 0.79, 0.69, 0.71 0.81, 0.70, 0.74 0.84, 0.71, 0.76	0.73, 0.71, 0.71 0.78, 0.73, 0.75 **0.82, 0.77, 0.79** **0.84, 0.82, 0.82**

*r is recall, p is precision, and f1 is F1-score. **“Other” includes declined races.

For error analysis, a manual audit of a small sampling of cases from each corpus with false negatives for positive mentions of “**fever**” (in the N3C COVID corpus) and “**diarrhea**” (in the UMN PASC corpus) are shown in Table 7. BioMedICUS had a false negative rate of 0.28, 0.44 and 0.55 respectively for “**fever**,” “**diarrhea**” and “**chest pain**” while both the LLMs had a FNR of 0.54, 0.22 and 0.88 for “**fever**,” “**diarrhea**” and “**chest pain**”, respectively.

**Table 7:** Representative examples of false negatives for positive mentions of “fever” in N3C COVID corpus, “diarrhea” in UMN PASC corpus and “chest pain” in N3C corpus as returned by BioMedICUS and both LLMs along with explanations.

Corpus/ Symptom	Ground Truth	BioMedICUS	LLMs’	Issues
N3C COVID/Fever	1	0	0	both UMLS and LLM did not pick up 38 °C’ following heading of ‘Temp’ for positive mention; other similar
	1	1	0	contextual: “had been sick with fever... over 12 days prior to his admission.”, etc.
	1	0	1	UMLS did not pick up “increase in temperature” for positive mention; “highest temp 37. 5,” etc.
UMN PASC/Diarrhea	1	0	0	classified as negation: “Mild diarrhea today, kaopectate resolved”
	1	1	0	none
	1	0	1	improper negation: “Constipation - senna not doing well, still either rabbit turds or diarrhea”
N3C COVID/Chest Pain	1	0	0	missed lexical synonym: “Persistent pain or pressure in the chest,” etc.
	1	1	0	missed case of both positive and negative mention due to “possible” symptom
	1	0	1	none

### Fine-tuned LLaMA2-13B vs un-fine-tuned LLaMA2-13B

#### Performance of un-fine-tuned LLaMA2-13B.

Despite using the same prompt structure consistently, the un-fine-tuned model generated highly variable outputs. Three major challenges were observed: (1) Inconsistent Output Formatting- While some outputs adhered to the requested JSON format, others generated free-text narratives that required extensive parsing; (2) Terminology Variability- The model frequently produced synonymous terms rather than the predefined symptom categories (e.g., *“****difficulty taking a deep breath****”* instead of *“****shortness of breath****”*, *“****memory loss****”* instead of *“****mental status change****”*; (3) Misinterpretation of Prompt Instructions- In some instances, the model reversed the classification logic, labeling symptoms as present when they were absent and vice versa. These inconsistencies rendered direct accuracy computation impractical. Instead, a manual review of 100 clinical notes showed that only 77% of outputs were structurally consistent, and 38% of cases included symptoms beyond our predefined scope, necessitating extensive post-processing.

#### Performance of fine-tuned LLaMA2-13B.

Fine-tuning the LLaMA2-13B model on clinician-annotated data substantially improved output structure and reliability. The fine-tuned model consistently produced structured outputs in a tensor format of shape (14, 3), where each row corresponded to a symptom and columns represented the probability scores for present (1), absent (0), or negated (-1). This structured output eliminated the need for manual correction and facilitated seamless integration into clinical workflows. The fine-tuned model further addressed the symptom mapping issue through: (1) Pre-training knowledge- The model leveraged prior exposure to medical terminology, improving its understanding of symptom equivalencies; (2) Expert-annotated training data- Symptoms were consistently mapped to standardized terms (e.g., *“****SOB****”* and *“****dyspnea****”* were labeled as *“****shortness of breath****”*). A manual assessment of structured output consistency confirmed that 100% of fine-tuned model outputs adhered to the expected structure, compared to 77% for the un-fine-tuned model. Additionally, the fine-tuned model restricted predictions to the predefined symptom list, eliminating extraneous outputs (Table 8).

**Table 8:** Example outputs of fine-tuned and un-fine-tuned LLaMA2-13B model.

Input Clinical Note*	Fine-Tuned Model Output (Structured Labels)	Un-Fine-Tuned Model Output (Free-Text)
Clininical_note_1.txt	{ bone or joint pain: 0 mental status changes: -1 chest pain/palpitations: 0 cough: -1 diarrhea: 0 shortness of breath: 1 fatigue: 0 fever: -1 headaches: 0 changes in sleep: 0 nausea/vomiting: 0 nasal congestion: 0 sore throat: 0 loss of smell and taste: 1 }	“ Symptoms = {“Positive”: [“shortness of breath”, “difficulty taking a deep breath”, “feeling like she can’t take a full breath”, “chills”, “head felt heavy”, “loss of taste”], “Negative”: [“fever greater than 100 (patient denies having a fever)”, “cough (patient denies having cough)”, “Breathing problems (patient denies having breathing problems, but mentions difficulty taking a deep breath)”, “ICU or delirium during hospitalization (patient denies experiencing these)”] } ”
Clinical_note_2.txt	{ bone or joint pain: 1 mental status changes: 0 chest pain/palpitations: -1 cough: 0 diarrhea: 0 shortness of breath: -1 fatigue: -1 fever: 0 headaches: 0 changes in sleep: 0 nausea/vomiting: 0 nasal congestion: 0 sore throat: 0 loss of smell and taste: 1 }	“Positive Symptoms: *Neck and low back pain *Chronic low back pain *Chest Pain (denied) *Shortness of breath (denied) *Numbness, paresthesias, or *Weakness (denied) Negative Symptoms: *Numbness *Paresthesias *Weakness *Chest Pain *Shortness of breath *Recent travels”

* Actual clinical text can’t be shown due to protected health information

**Fig 1:**
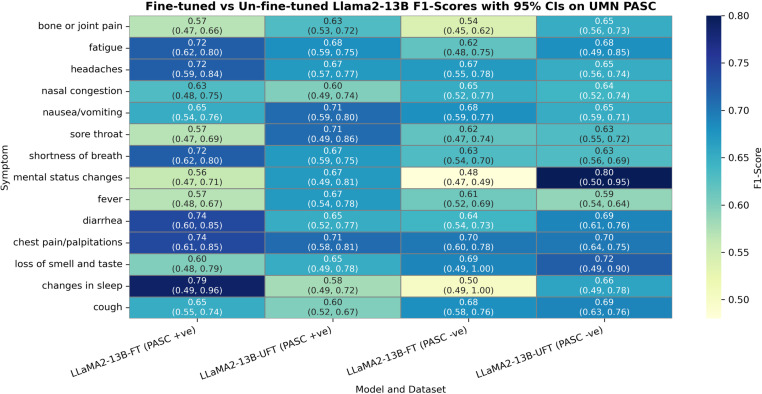
Comparison of macro-averaged F1-scores of fine-tuned (FT) and un-fine-tuned (UFT) Llama2-13B models on UMN PASC positive ( +ve) and negative (-ve) symptom mentions.

As displayed in Table 8, the outputs of the fine-tuned model are always consistent in their structure making the fine-tuned model suitable for direct integration into clinical workflows, while the output of un-fine-tuned model is highly variable in its structure and thus requires extensive post-processing before use, making it impractical for deployment. After manually fixing the output predictions of the un-fine-tuned LLaMA2-13B model, we compared the symptom classification performance of the fine-tuned and un-fine-tuned LLaMA2-13B model. Fig 1 displays symptom-wise macro-average F1-scores with 95% CIs calculated over UMN PASC positive and negative symptom mentions.

The performance comparison between the fine-tuned and un-fine-tuned LLaMA2-13B models on the symptom classification task on UMN PASC reveals nuanced differences in their predictive capabilities. For UMN PASC-positive cases, the fine-tuned model generally exhibited competitive or superior performance for symptoms such as **chest pain/palpitations** (F1-score: 0.74 vs. 0.71), **diarrhea** (F1-score: 0.74 vs. 0.65), and **shortness of breath** (F1-score: 0.72 vs. 0.67), suggesting that fine-tuning enhanced the model’s ability to capture these specific symptoms. Notably, the fine-tuned model also showed marked improvement in classifying **changes in sleep** (F1: 0.79 vs. 0.58), indicating its sensitivity to less common but clinically relevant features. However, the un-fine-tuned model outperformed the fine-tuned variant for **mental status changes** (F1-score: 0.67 vs. 0.56) and **nausea/vomiting** (F1-score: 0.71 vs. 0.65), possibly due to the latter’s stronger reliance on pre-existing knowledge for these symptoms. For UMN PASC-negative cases, the differences were less pronounced, though the fine-tuned model demonstrated slight advantages for **cough** (F1-score: 0.68 vs. 0.69) and **nasal congestion** (F1-score: 0.65 vs. 0.64), while the un-fine-tuned model performed better for **loss of smell and taste** (F1-score: 0.72 vs. 0.69). Overall, fine-tuning appears to offer targeted improvements for certain symptoms, particularly those with higher clinical specificity, but may not uniformly enhance performance across all categories.

## Discussion

This study aimed to fine-tune LLMs (LLaMA2-13B and LLaMA3-8B) for extraction of patient symptoms from unstructured clinical notes and demonstrate its comparable effectiveness to rule-based IE methods. The focus was on assessing the models’ performance across multiple datasets, their handling of both positive and negative symptom mentions, and the fairness of the models across demographic groups. The study also aimed to analyze the prevalence of symptoms across different corpora and perform an error analysis to identify the key challenges faced by these models. The major findings of this study are as follows: (1) BioMedICUS showed higher recall for certain symptoms, while the LLMs excelled in precision and F1-scores for others, while both had similar performance for some symptoms, specifically, LLaMA3-8B showed superior performance in detecting negative symptom mentions, outperforming both BioMedICUS and LLaMA2-13B in many cases; (2) The LLMs, specifically LLaMA2-13B had generalizability and bias issues due to overfitting from training on a non-diverse dataset and single annotator bias, highlighting the need for diverse, high-quality datasets and robust annotations; (3) Significant differences in symptom distribution were observed across the different corpora used in the study, as highlighted by the prevalence analysis; (4) Manual audits revealed higher FN for BioMedICUS and higher false positives (FP) for the LLMs, often due to misinterpreting context or section headers.

### Performance evaluation

Comparing the generalizability of the fine-tuned LLMs and BioMedICUS for extracting symptoms from clinical notes reveals key differences. BioMedICUS, which uses techniques like “normalized bag of words” and UMLS, shows high generalizability due to its universal rules not limited by specific corpora, effectively identifying symptoms across diverse datasets. In contrast, the LLMs’ performance is closely tied to its training data, mostly from UMN PASC patients, with only 12% from UMN COVID patients. Consequently, the LLMs performed similarly to BioMedICUS on the UMN PASC hold-out test set but underperformed on the acute N3C COVID test set, highlighting issues of overfitting and inadequate acute COVID-19 patient representation in the training data. Additionally, the LLMs’ UMN COVID training labels, annotated by a single annotator without inter-annotator agreement, likely reduced its performance. These findings suggest that while LLMs offer flexibility in capturing nuanced symptoms, their generalizability can be improved with well-annotated datasets, regularization, and robust prompt engineering.

We found that for positive mentions of UMN PASC symptoms, choosing between LLMs and BioMedICUS depends on priorities: BioMedICUS prioritizes maximizing identification with higher recall, while the LLMs prioritize minimizing FPs, resulting in better precision. For negative mentions, while BioMedICUS appears more robust across a broad range of symptoms, LLMs’ strengths in specific areas suggest its value lies in targeted symptom identification. For N3C COVID symptoms, BioMedICUS consistently outperformed the LLMs, specifically LLaMA2-13B, across most positive and negative symptom mentions, indicating its ability to generalize knowledge to new data.

One of the standout findings was LLaMA3–8B’s superior ability to detect negative symptom mentions, where it consistently outperformed both LLaMA2-13B and BioMedICUS. LLaMALLaMA3–8B’s strength in negative mention detection could be due to its advanced language modeling capabilities, allowing it to better understand the complex syntactic structures and negations common in clinical texts.

Our results align with experiments performed by Patra, *et al*. who compared the performance of rule-based systems with LLMs for extracting information from clinical psychiatry notes. They found that the rule-based systems outperformed the LLMs across most of their cases [27]. Our findings are further supported by Chen, *et al.*, who compared LLMs like GPT-3.5 and GPT-4 [28] against fine-tuned BERT and BART [29] models for biomedical natural language processing applications. They found that GPT-4’s performance was competitive or better than the fine-tuned BERT and BART models in 6 out of 12 benchmarks especially for reasoning and generative tasks [30].

### Prevalence analysis

S1 Table highlights the differences in the ranking of symptoms by prevalence and TF-IDF in various corpora. For example, the symptom “**fever**” is ranked first by TF-IDF in N3C COVID, but it ranks second and fourth in the UMN COVID and UMN PASC corpora, respectively. The number of times “**fever**” appears in these corpora differs even more. Symptom mentions of “**diarrhea**” do not appear among top rankings of UMN PASC by TF-IDF. Additionally, in N3C COVID “**problem with smell or taste**” was not among the top 10 when ranked by prevalence but was seventh when ranked by TF-IDF. Lastly, “**chest pain**” appears only in UMN COVID. Results of the ANOVA test confirm that both structure and content of these corpora significantly influence the TF-IDF scores. This is likely because the corpora are organized differently or contain different information. Moreover, the large effect sizes (η² = 0.52 for corpora and η² = 0.35 for symptoms) show that both the corpus format and the presence or absence of symptoms have a substantial influence on the TF-IDF score distribution across the corpora. This highlights how TF-IDF can identify symptoms that, while less common, are more characteristic of a condition, such as “problem with smell or taste” in COVID. Ranking by TF-IDF emphasizes the uniqueness of certain symptoms, which may be underrepresented by prevalence alone, offering deeper insights for diagnosis and treatment. These factors and their effect on model training and extraction performance warrant further examination, especially with respect to model generalization.

### Error analysis

As shown in Table 7, for the N3C COVID corpus, both BioMedICUS and the LLMs missed various terms for “**fever**” not present in either the UMLS or the LLMs’ lexicon. Additionally, the LLMs overlooked contextual sentences ending with “**had been sick**”. However, while the UMLS lexicon missed terms like “**increase in temperature**”, the LLMs detected them, demonstrating its sensitivity to such phrases due to its pre-training on general English. For “**diarrhea**”, both BioMedICUS and the LLMs incorrectly negated sentences containing “**diarrhea**” that ended with “**resolved**” missing the temporality of these mentions. However, the LLMs correctly contextualized a negating phrase unrelated to having “**diarrhea**”, whereas BioMedICUS did not. For “**chest pain**”, both classifiers missed terms not found in the UMLS or the LLMs’ lexicon, and the LLMs failed to classify terms with both positive and negative mentions of “**chest pain**” accurately.

For both the LLMs and BioMedICUS various symptoms had been mislabeled as FP due to neither using section header detection to filter out irrelevant mentions from notes. On the other hand, certain templated sections within clinical notes were correctly identified by LLMs but not by BioMedICUS. Consequently, we noted a significant difference between RTF structured and unstructured versions of the UMN PASC corpus using BioMedICUS’ rule-based selection header detection: In RTF notes, symptoms under “HPI” were identified 785 times compared to 424 times in unstructured notes, suggesting that RTF’s structure aids in distinguishing relevant section headers, with potential for reducing FP.

## Limitations

The limitations of our study include: (1) The LLaMA2-13B and LLaMA3-8B models were fine-tuned on a relatively small and non-diverse dataset, primarily sourced from the UMN PASC corpus, which may have contributed to issues of overfitting and limited generalizability to other datasets, such as the N3C COVID corpus. The use of a single annotator for certain parts of the training data (UMN COVID) further introduced potential bias, limiting the robustness of the models. Inter-annotator agreement was not consistently applied, which could have reduced the consistency and quality of the ground truth labels. (2) The demographic fairness analysis revealed that the LLaMA models, particularly LLaMA2-13B, struggled with equity across different patient populations. While LLaMA3-8B showed improvements, both LLMs exhibited performance disparities when evaluated on gender and race, highlighting the challenge of ensuring fairness in machine learning models trained on imbalanced datasets. (3) The handling of negations and contextual nuances, while improved in LLaMA3-8B, still posed a challenge for both the rule-based and LLM systems. The error analysis demonstrated that both systems missed or misclassified important symptoms due to difficulties in understanding complex linguistic structures, especially when symptoms were negated or temporally referenced. (4) the absence of structured section headers in some clinical notes, particularly those not in rich text format (RTF), led to missed symptom detections, especially in BioMedICUS. The reliance on text formatting limits the general applicability of both the rule-based and LLM systems across different types of clinical documentation. (5) Lexica used in this study may be incomplete thus leading to an increase in FN across some symptoms. (6) Length of notes: some notes might be too large to be processed by our fine-tuned LLMs. In such cases, the notes must be processed in chunks, which slows down the process. (7) A key limitation of using ANOVA with TF-IDF as the dependent variable is that it analyzes differences in the feature itself (TF-IDF values) rather than evaluating the real-world performance of models that use these features. While ANOVA can identify significant variance in TF-IDF across groups, such as different corpora or symptoms, it does not provide insight into whether these differences lead to better outcomes in tasks like classification or information retrieval.

## Conclusion

This study highlights the significant issues of model bias and generalizability in our fine-tuned LLMs, specifically in fine-tuned LLaMA2-13B, due to demographic variability and corpus differences. Our evaluation revealed that the LLMs, trained predominantly on UMN PASC patients with limited acute COVID-19 representation, showed overfitting and insufficient generalization, potentially exacerbated by single annotator bias. These findings provide real-world evidence of the challenges LLMs may face, underscoring the necessity for diverse, high-quality training datasets and robust annotation processes to mitigate bias and enhance generalizability. Our findings also indicated that the rule-based approach and the LLM approach each have strengths and limitations in performing IE tasks applied to clinical NLP.

While this study focused on PASC, the implications of our findings extend beyond this specific condition. The IE techniques using LLMs developed in this research can be adapted and applied to other complex illnesses with diverse symptomatology, such as chronic fatigue syndrome, fibromyalgia, or autoimmune disorders. This broader application underscores the potential of LLMs in advancing clinical NLP across various medical domains, while also emphasizing the need for careful consideration of bias and generalizability in each new application.

## Supporting information

S1 File S1 Table. Symptoms ranked by TF-IDF and prevalence. **S2 Table.** Example ANOVA analysis data input. **S3 Table.** Simulated primary data frame. **S4 Table.** Analysis of variance (ANOVA) results. **S5 Table.** Tukey’s honest significant difference (HSD) test. **S6 Table.** Magnitude Analysis of the effects of corpus and symptom on log-transformed TF-IDF values.
(DOCX)
