# Supplementary material for: A comparative analysis of large language models versus traditional information extraction methods for real-world evidence of patient symptomatology in acute and post-acute sequelae of SARS-CoV-2
Source: PLoS One. 2025 May 15;20(5):e0323535. doi: 10.1371/journal.pone.0323535 (PMC12080813; doi:10.1371/journal.pone.0323535)
Supplement: S1 File — Symptoms ranked by TF-IDF and prevalence. S2 Table. Example ANOVA analysis data input. S3 Table. Simulated primary data frame. S4 Table. Analysis of variance (ANOVA) results. S5 Table. Tukey’s honest significant difference (HSD) test. S6 Table. Magnitude Analysis of the effects of corpus and symptom on log-transformed TF-IDF values. (DOCX) [file pone.0323535.s001.docx]

**Supporting Information**

**Prevalence analysis**

**S1 Table. Top 10 normalized symptoms ranked by TF-IDF and prevalence. Prevalence within each corpus noted*.**

| **UMN PASC TF-IDF** | **UMN PASC Prevalence** | **UMN COVID TF-IDF** | **UMN COVID Prevalence** | **N3C COVID TF-IDF** | **N3C COVID Prevalence** |
| --- | --- | --- | --- | --- | --- |
| pain (0.073) | pain (0.073) | shortness of breath (0.118) | fever (0.119) | fever (0.059) | pain (0.090) |
| cough (0.043) | anxiety (0.047) | fever (0.119) | shortness of breath (0.118) | pain (0.090) | shortness of breath (0.084) |
| shortness of breath (0.044) | shortness of breath (0.044) | cough (0.100) | cough (0.100) | nausea or vomiting (0.064) | nausea or vomiting (0.064) |
| fever (0.028) | cough (0.043) | nausea or vomiting (0.058) | nausea or vomiting (0.058) | shortness of breath (0.084) | fever (0.059) |
| wheezing (0.011) | depression (0.036) | bone or joint pain (0.053) | bone or joint pain (0.053) | cough (0.053) | cough (0.053) |
| anxiety (0.047) | fatigue (0.034) | wheezing (0.012) | fatigue (0.041) | dizziness or vertigo (0.045) | dizziness or vertigo (0.045) |
| fatigue (0.034) | nausea or vomiting (0.030) | chest Pain (0.034) | chest pain (0.034) | problem with smell or taste (0.021) | diarrhea (0.040) |
| bone or joint pain (0.030) | bone or joint pain (0.030) | peripheral edema (0.023) | diarrhea (0.031) | chills (0.014) | abdominal pain (0.039) |
| nausea or vomiting (0.030) | headaches (0.029) | headaches (0.023) | peripheral edema (0.023) | bone or joint pain (0.024) | respiratory depression (0.037) |
| headaches (0.029) | fever (0.028) | diarrhea (0.031) | chills (0.023) | diarrhea (0.040) | fatigue (0.034) |

*The number in parentheses is the prevalence normalized to the number of symptoms

**S2 Table. Example ANOVA Analysis Data Input**

| **Symptom** | **Corpus** | **Log-TFDF** |
| --- | --- | --- |
| shortness of breath | clarity_covid | 0.343132 |
| fever | clarity_covid | 0.311289 |
| cough | clarity_covid | 0.145626 |
| nausea and or vomiting | clarity_covid | 0.032073 |
| myalgia | clarity_covid | 0.010954 |

**S3 Table. Simulated primary data frame**

| **Start UMLS Concept** | **End UMLS Concept** | **Mention** | **Note ID** | **Polarity** | **TUI** | **Key Norm** | **Header** | **Sentence** |
| --- | --- | --- | --- | --- | --- | --- | --- | --- |
| 104 | 123 | Respiratory failure | s5111111 | 1 | T047 | Respiratory Failure | Indication | Out from surgery, in respiratory failure |

**S4 Table. Analysis of Variance (ANOVA) results evaluating the effects of Corpus and Symptom on TF-IDF values, with both code used for analysis and relevant test output.**

|  | **sum_sq** | **df** | **F** | **PR (>F)** |
| --- | --- | --- | --- | --- |
| C (corpus) | 266.02 | 2.00 | 305.78 | 4.79e-55 |
| C (symptom) | 177.58 | 79.00 | 5.17 | 9.87e-19 |
| Residual | 68.72 | 158.00 | NaN | NaN |

**S5 Table. Tukey's honest significant difference (HSD) test comparing log-transformed TF-IDF values across different corpora, with both code used for analysis and relevant test output.**

| **Multiple comparisons of means - Tukey HSD, FWER=0.05** | | | | | | |
| --- | --- | --- | --- | --- | --- | --- |
| **group1** | **group2** | **meandiff** | **p-adj** | **lower** | **upper** | **reject** |
| UMN COVID | UMN PASC | 2.57 | 0.00 | 2.19 | 2.95 | True |
| UMN COVID | N3C COVID | 1.06 | 0.00 | 1.44 | 1.44 | True |
| UMN PASC | N3C COVID | -1.51 | 0.00 | -1.89 | -1.13 | True |

**S6 Table. Magnitude Analysis of the Effects of Corpus and Symptom on Log-Transformed TF-IDF Values Using Eta Squared (η²), with both code used for analysis and relevant test output.**

|  | **sum_sq** | **df** | **F** | **PR (>F)** | **eta_sq** |
| --- | --- | --- | --- | --- | --- |
| corpus | 266.02 | 2.00 | 305.78 | 4.79e-55 | 0.52 |
| symptom | 177.58 | 79 | 5.17 | 9.87e-19 | 0.35 |
| residual | 68.73 | 158.00 | NaN | NaN | 0.13 |

**BioMedICUS/Microservice text analysis platform (MTAP) concept extraction**

The BioMedICUS/MTAP pipeline incorporates: (1) Sentence boundary detection using a pre-trained bi-LSTM model [(7)](https://www.zotero.org/google-docs/?DSJl4G) (2) Rule-based matching for section header detection [(30)](https://www.zotero.org/google-docs/?Z95eo6) (3) Concept labeling using various matching methods, including normalized bag-of-words matching against UMLS terms [(31)](https://www.zotero.org/google-docs/?DSGsUE), and (4) Negation detection using NegEx [(32)](https://www.zotero.org/google-docs/?SbLTXo).

**Lexica**

This study utilized four lexica: (1) A lexicon of acute COVID-19 symptoms as discussed in Silvermal, *et al.* based on guidelines provided by the Centers for Disease Control and Prevention (CDC) [(2,33)](https://www.zotero.org/google-docs/?9cHv4j); (2) A lexicon of PASC symptoms based on CDC (2022) guidelines and [(34)](https://www.zotero.org/google-docs/?ev24qE); (3) A lexicon of acute COVID-19 symptoms developed by the N3C consortium [(21)](https://www.zotero.org/google-docs/?t7Jp4I); and (4) PASCLex developed by [(6)](https://www.zotero.org/google-docs/?1TIs6T). These lexica were normalized using PASCLex to allow for mappings between each corpus (please see <https://shorturl.at/iqBC3> for normalized mappings between lexica used in this study).

**TF-IDF Code**

import pandas as pd

from sklearn.feature_extraction.text import TfidfVectorizer

df = pd.read_csv(r"b9_vns_cleaned_symptoms.csv")

df = df.rename(columns={'key_norm':'symptoms'})

df['symptoms'] = df['symptoms'].fillna("None").astype(str)

# Replace spaces with underscores in the symptoms

df['symptoms'] = df['symptoms'].str.replace(' ', '_')

df['symptoms'] = df['symptoms'].str.replace('/', '_')

# Aggregate symptoms by note_id

grouped_df = df.groupby('note_id')['symptoms'].apply(lambda x: ' '.join(x)).reset_index()

# Vectorize the symptoms using TfidfVectorizer

vectorizer = TfidfVectorizer(token_pattern=r'(?u)\b\w+\b')

#Calculate the TF-IDF scores for each note id (grouped symptoms)

tfidf_matrix = vectorizer.fit_transform(grouped_df['symptoms'])

# Create a DataFrame from the matrix of TF-IDF scores

tfidf_df = pd.DataFrame(tfidf_matrix.toarray(), columns=vectorizer.get_feature_names_out(), index=grouped_df[ 'note_id'])

# Aggregate the TF-IDF scores by summing them up across all notes for each symptom

aggregate_tf_idf = tfidf_df.sum(axis=0).sort_values(ascending=False).reset_index()

# Rename the columns to 'symptom' and 'aggregate_tf_idf'

aggregate_tf_idf.columns = ['symptom', 'aggregate_tf_idf']

# Show the result

aggregate_tf_idf

Caption: This code aggregates symptom data by note id and computes TF-IDF scores to analyze the relative importance of each symptom within the context of the entire dataset. It begins by grouping symptoms by note ID, combining all symptoms for each note into a single document. The TF-IDF vectorizer is then applied to calculate the scores, resulting in a matrix where each row represents a note id and each column represents a symptom and its corresponding TF-IDF value. For example (0,40), 0.095 would represent note id 0, and symptom 40 has a TF-IDF of 0.095. For easier interpretability, the matrix was converted back to a pandas dataframe; each row corresponds to a note id and each column corresponds to a symptom (S7 Table). The final step adds up the TF-IDF for each symptom to get an overall value for all notes.

**S7 Table. Partial example of a row prior to aggregating for an overall TF-IDF.**

| **note_id** | **abdominal pain** | **agitation** | **anxiety** |
| --- | --- | --- | --- |
| 786 | 0.10 | 0.068 | 0.14 |

**ANOVA/Effect Size Code**

import pandas as pd

import numpy as np

from scipy import stats

import seaborn as sns

import matplotlib.pyplot as plt

from sklearn.preprocessing import StandardScaler

import matplotlib.pyplot as plt

import seaborn as sns

import statsmodels.api as sm

from statsmodels.formula.api import ols

from statsmodels.stats.multicomp import pairwise_tukeyhsd

from statsmodels.multivariate.manova import MANOVA

from scipy.stats import zscore

import statsmodels.api as sm

from statsmodels.formula.api import ols

df1 = pd.read_csv('b9_clarity_covid_w_headers_tf-idf.csv')

df2 = pd.read_csv('b9_clarity_pasc_w_headers_corrected_tf_idf.csv')

df3 = pd.read_csv('b9_mayo_covid_w_headers_tf-idf.csv')

merged_df = df1.merge(df2, on='symptom', how='outer', suffixes=('_df1', '_df2'))

merged_df = merged_df.merge(df3, on='symptom', how='outer', suffixes=('', '_df3'))

merged_df = merged_df.rename(columns={

'aggregate_tf_idf_df1':'clarity_covid',

'aggregate_tf_idf_df2':'clarity_pasc',

'aggregate_tf_idf': 'mayo_covid'

})

# Plot histograms and QQ-plots for each corpus

for column in ['clarity_covid', 'clarity_pasc', 'mayo_covid']:

plt.figure(figsize=(12, 5))

plt.subplot(1, 2, 1)

drop_df[column].hist(bins=20, alpha=0.7)

plt.title(f'Histogram of {column}')

plt.subplot(1, 2, 2)

stats.probplot(drop_df[column], dist="norm", plot=plt)

plt.title(f'QQ-Plot of {column}')

plt.tight_layout()

plt.show()

#reshape df

long_df = pd.melt(drop_df, id_vars=['symptom'], var_name='Corpus', value_name='TF_IDF')

long_df.head(10)

long_df['log_tfidf'] = np.log(long_df['TF_IDF'] + 1e-6)

# Checking normal after transformation

# Create plots for each corpus

for corpus in corpora:

# Filter the DataFrame by corpus

subset = long_df[long_df['Corpus'] == corpus]

# Create a figure with 2 subplots

plt.figure(figsize=(12, 6))

plt.suptitle(f'Data Analysis for {corpus}')

# Histogram for the weighted log values

plt.subplot(1, 2, 1)

plt.hist(subset['log_tfidf'], bins=5, color='blue', alpha=0.7)

plt.title('Histogram of Log TF-IDF')

plt.xlabel('Log TF-IDF')

plt.ylabel('Frequency')

# Q-Q plot for the weighted log values

plt.subplot(1, 2, 2)

stats.probplot(subset['log_tfidf'], dist="norm", plot=plt)

plt.title('Q-Q Plot of Log TF-IDF')

# Show the plot

plt.tight_layout(rect=[0, 0.03, 1, 0.95]) # Adjust layout to make room for the title

plt.show()

# Perform ANOVA

model = ols('log_tfidf ~ C(Corpus) + C(symptom)', data=long_df).fit()

anova_results = sm.stats.anova_lm(model, typ=2) # type 2 sum of squares is generally preferred

print(anova_results)

tukey = pairwise_tukeyhsd(endog=long_df['log_tfidf'], groups=long_df['Corpus'], alpha=0.05)

print(tukey)

# Perform ANOVA

model = ols('TF_IDF ~ C(Corpus) + C(symptom)', data=long_df).fit()

anova_results = sm.stats.anova_lm(model, typ=2) # Type 2 ANOVA DataFrame

print(anova_results)

# Tukey's HSD Test

tukey = pairwise_tukeyhsd(endog=long_df['TF_IDF'], groups=long_df['Corpus'], alpha=0.05)

print(tukey)

# Tukey's HSD Test

tukey = pairwise_tukeyhsd(endog=long_df['TF_IDF'], groups=long_df['symptom'], alpha=0.05)

print(tukey)

model = ols('log_tfidf ~ C(Corpus) + C(symptom)', data=long_df).fit()

anova_table = sm.stats.anova_lm(model, typ=2)

# Calculate Eta Squared for each factor

anova_table['eta_sq'] = anova_table['sum_sq']/sum(anova_table['sum_sq'])

print(anova_table)

The above code integrates three TF-IDF datasets by merging them based on symptoms. Then, the distribution of TF-IDF scores for each corpus was analyzed for normality through Q-Q plots. Due to non-normal distribution, a logarithmic transformation was applied and a Q-Q plot was reevaluated to check for normality. An ANOVA was then conducted to assess the impact of corpus and symptom on TF-IDF scores, followed by Tukey's HSD tests for detailed pairwise comparisons. Finally, the analysis computes Eta squared values to quantify the effect sizes to determine which factors contribute to the variance in TF-IDF scores.
